# QAnon shifts into the mainstream, remains a far-right ally

**DOI:** 10.1016/j.heliyon.2022.e08764

**Published:** 2022-01-21

**Authors:** Saifeldeen Zihiri, Gabriel Lima, Jiyoung Han, Meeyoung Cha, Wonjae Lee

**Affiliations:** a Yale Law School, New Haven, CT 06520, United States; b School of Computing, KAIST, Daejeon 34141, Republic of Korea; c Data Science Group, Institute for Basic Science (IBS), Daejeon 34126, Republic of Korea; d Moon Soul Graduate School of Future Strategy, KAIST, Daejeon 34141, Republic of Korea; e Graduate School of Culture Technology, KAIST, Daejeon 34141, Republic of Korea

**Keywords:** QAnon, Conspiracy theory, Extremist groups, Far-right, Far-left, Social media, Telegram, Extremism

## Abstract

The rise of domestic fringe groups within the United States has been well documented, threatening political and social stability. The QAnon conspiracy theory has developed as one such destructive group, though it remains a largely misunderstood movement. Through a mixed-methods analysis of over 3.5 million messages on Telegram from three politically extreme communities - QAnon, far-right, and far-left - we studied how QAnon fits within the larger non-mainstream political ecosystem. Our analysis provides insights into how this new political movement is dissimilar to the far-right or the far-left but shares offline interests with the far-right. The topics discussed within QAnon communities were unique to the movement and the least reactive to news cycles. Links shared by QAnon, particularly from YouTube and Twitter, were often from traditional conservative sources and individuals, whereas the far-left and far-right relied on less mainstream sources. Finally, though QAnon may be distinct from the other communities, it coalesces with the far-right during particular political events where the former United States President Trump is a major player. Our findings highlight how fringe groups react to major political events and navigate conversations online.

## 1 Introduction

Conspiracy theories have historically been linked to various negative impacts of tribalism, whether it be prejudice, violence against those considered the out-group, or social harm [1]. Yet, not all conspiracy theories are made equal. Certain theories prove uniquely insidious, targeting people's trust in both out-groups and the political institutions designed to mitigate and reconcile conflict [2]. Belief in this genre of conspiracy theories is often associated with endorsements of political violence [3], playing an important social and functional role for extremist movements. The acceptance of alienating conspiracy theories can thus establish extremist groups and radicalize them into violence. Researchers have identified three ways in which this occurs. First, establishing enemies with which the group can define itself against. Second, delegitimizing voices of dissent both within and outside of the movement. Finally, symbolizing violence as necessary to “awaken” people from their proverbial slumber [2]. Within the United States, the relationship between conspiracy theories and political extremism has been identified across the political spectrum, with members of both the political far-right and far-left being most susceptible to these theories [4]. The development of the particularly alienating QAnon conspiracy theory, and its subsequent growth into a global movement, should thus raise a significant alarm.

QAnon has already showcased a propensity for harm, its growth in the online space having manifested into tangible offline harms. A series of criminal activities have been linked to its believers leading to acts of armed violence [5]. The most notable action has been the pro-Trump mob attack of the United States (US) Capitol on January 6, 2021, in which many members were confirmed QAnon supporters [6]. Yet, QAnon alone does not fully account for the harm that occurred that day [7]. Previous literature has highlighted that protestors “come together […] when they know where to go and why, when they expect others to join them, and when they expect their peers to judge them favorably” [8]. It is thus crucial to observe the interaction between QAnon and other extreme political movements to understand the January mob attack better. Past research on QAnon has primarily focused on its political and cultural characteristics driving violent behaviors, and little attention has been paid to whether (and how) QAnon may be aligned with other parties that make way for them. Here, we offer an analysis of three extreme political communities (i.e., QAnon, the far-left, and far-right) and study how they may be aligned concerning a series of offline social and political events in 2020 and 2021. We investigate the relationship between major political events and the response of fringe groups on the messaging platform Telegram, attempting to observe how QAnon converses around these events and its similarity with other politically extreme movements.

The QAnon conspiracy theory first emerged as an Internet phenomenon in October 2017 on the imageboard 4chan. An anonymous poster claiming to be a high-level government insider, later named QAnon, wrote a series of cryptic posts on various aspects of US domestic and foreign affairs [9]. Often written in the form of a question, these posts–known as Q drops–circulated rapidly via anonymous imageboards. In aggregate, there have been 4,953 Q drops over the past three years, with the final message to date being posted December 8, 2020 on the website 8kun [10]. Conspiracy theorists believe that Q drops offer insight into the true global order and their cryptic style serves to protect QAnon from the alleged enemies that have gained control over institutional power [11]. The identity of QAnon has also been expanding; at times, online posts have implied that more than one person is involved with the account, and more specifically, that former US President Trump may be involved with QAnon [12].

The exact nuances comprising the conspiracy are challenging to pinpoint. The theory posits that a range of political, social, and cultural elites are waging war against freedom and decency while Trump leads a counterattack against these malicious forces [13]. Adherents are expected to conduct their research and connect the dots themselves, allowing the community to associate events, like the fire at Notre Dame Cathedral in April of 2019, to the broader ideological framework [14]. The conspiracy has grown to be a global movement, and different international localities have developed their subculture. For instance, the foothold in Japan has a particular fascination with Michael Flynn that is not found in the German QAnon community [15]. As a result of the wide range of conspiracies that develop and spread both from Q drops and QAnon adherents, individuals can recognize and believe in each conspiracy without identifying as a QAnon supporter or even being aware of the overarching movement [16]. The most salient example of this phenomenon can be seen in various widely spread myths surrounding child trafficking that were later discovered to be recruitment efforts for the QAnon movement [17].

Those who propagate and subscribe to QAnon beliefs do not often fit within a particular ideological mold. QAnon “appears to find support among both the political right and left,” with “the extremity of political orientation” mattering far more than political leaning [18]. While members of the alternative right (alt-right) have played a role in the early expansion of the conspiracy [19], other QAnon members have been described as “casual conspiracy theorists” involved with the movement “for the fun of the immersive game and the community more than the conspiracy or sense of regaining control” [20]. Outside of politics, contributors to the movement range substantially in their career choices, from celebrities and politicians to men's rights activists [20]. Much has also been highlighted regarding the support for QAnon among white evangelical Christians, with some surveys finding that as many as 27% of the religious group largely agrees with core QAnon beliefs [21]. It is important to note that researchers have criticized surveys that claim to uncover the percentage of support for QAnon among white evangelical Christians or the general American population more broadly. Pollsters have described the unique challenge of polling for QAnon support, finding that respondents can often be insincere, giving answers they believe are funny, on the opposite end, or socially desirable [22].

Another challenge is that QAnon's loosely organized network of beliefs makes it complex to place ideologically. Researchers have characterized the beliefs as far-right [23], right-wing with anti-Semitic and anti-LGBTQ elements [24], or simply a big tent conspiracy theory [25]. These perceptions of QAnon are not necessarily discordant, given the nature of data and events researchers have analyzed. Previous research has primarily analyzed the QAnon movement under the lens of other frameworks, whether it be far-right [26] or the appeal of conspiracy theories [27]. However, as the threat from QAnon has expanded, research has begun exploring the movement and its followers under their own merit.

Given that QAnon is a primarily online movement, scholars have focused on specific platforms. Smith, for instance, analyzed the QAnon movement from June 2018 to February 2020 on Twitter. He found that the community has become autonomous from the mainstream right-wing groups, though QAnon accounts are hard to distinguish from the Trumpian movement [28]. Looking at YouTube, Miller collected 26,000 video comments to QAnon-related content and observed that the fringe community produced an extreme right-wing foreign policy perspective [9]. On Reddit, Aliapoulios et al. found that links to Q drop aggregation sites appeared primarily in the right-wing oriented communities [29]. Surprisingly, relatively little research on QAnon examined Telegram, even though Telegram's use by fringe communities, particularly those less organized and more radical, has been well documented [30].

Telegram's appeal for fringe communities can be explained by its ability to reconcile private messaging and broadcasting, offering users both protected messaging and public channels to attain publicity [31]. Unlike other social media platforms that are public-facing and require aliases to maintain anonymity, Telegram is at its core an instant messaging app with an emphasis on privacy [30]. Privacy functions include the ability to immediately delete any messages sent or received, an anonymous forwarding option that prevents individuals from accessing the original sender's account, encrypted messages, and the ability to make group chats and broadcasting channels private. In particular, Telegram channels allow creators to send messages to an unlimited number of subscribers without sacrificing their privacy, an important functionality for fringe groups building up followers.

Past research studying QAnon on Telegram has demonstrated the movement's growth on the app. Garry et al. analyzed QAnon communities on Telegram and Gab and observed a 17% increase in group size over 39 days in 2020 [32]. Similarly, Walter and McCoy analyzed 125 Telegram political channels and reported that 64.8% of them grew in size over the studied week in 2020. Their qualitative assessment also suggests that QAnon groups acted as a source of Covid-19 disinformation and conspiracy theories [33]. Existing Telegram QAnon research emphasizes descriptive characteristics rather than content and often does not include an analysis of link sharing that happens within the movement. Likewise, existing research rarely contextualizes QAnon within other political extremist groups. Our research serves to bridge this gap and characterizes the QAnon movement within the fringe US political landscape.

## 2 Methods

For comparison with other extremist movements, we first defined far-left and far-right communities based on previous literature.^2^ Far-left communities are those that believe that people are trapped by the oppressive and dehumanizing institutions of either capitalism or structural racism [46]. To fall under this category, the far-left communities also need to believe in people's liberation through either protest or violent revolution. We excluded those groups that believed in electoral politics as a primary means of change, meaning that mainstream progressive political parties were excluded. This belief was determined by the information provided in the communities' descriptions and a qualitative assessment of the communities' conversations. This was done to lessen the influence of center and center-left parties and provide a more apparent distinction between the extreme left-wing communities and the other two communities analyzed.

We defined far-right communities as those groups that emphasized cultural homogeneity juxtaposed against the interests of minority groups within the United States [47]. Like our definition of far-left communities, we excluded those groups that explicitly believed in electoral politics as a primary means of change. We then defined QAnon communities as those that explicitly had either some iteration of QAnon in the title or the description or utilized a commonly used phrase within the QAnon community within the title or the description. Specific communities straddled the line between right-wing and QAnon, but we excluded those communities from our analysis.

Telegram provides two different communicative functions for members. The first is groups, which allow its members to participate in a conversation. The second is channels, which serve as a broadcasting tool for large audiences. Admins of each channel can determine whether subscribers can comment on channel posts. Both groups and channels are important information functions, and hence we do not discriminate between the two and include both in the analysis. We also included comments posted by subscribers in channels that allowed them. Our analyses did not cover images or videos shared within Telegram communities.

To find communities on Telegram, we employed the methodological sources of previous research. First, we attempted several searches in popular search engines outside of Telegram to determine the existence of large Telegram communities that had merited media attention. We then turned to the Telegram channels by examining common phrases. We also utilized a publicly available list of 747,262 Telegram Invite URLs from the Internet Archive [48], narrowing down searches based on popular QAnon, far-left, and far-right phrases. For QAnon communities, this included phrases such as “QAnon” and “WWG1WGA.” Finally, links to other Telegram communities were often shared within the various groups and channels. We randomly selected some of these communities for our analysis. This decision aimed to control for imbalances in the dataset as many of these communities would be too similar and most of these links were shared within the far-right or QAnon groups. We aimed to obtain a balanced dataset concerning political orientation.

We conducted a mixed-methods analysis of over 3.5 million messages shared by 171,114 accounts on 94 Telegram communities. After compiling 11 groups and 17 channels related to QAnon between January 1, 2020, and March 31, 2021, we compared their discourse to 61 channels and 6 groups belonging to far-right and far-left circles. Table 1 presents descriptive statistics of our dataset. We do not include any community that could be categorized as more than one type to remove ambiguity between the different extremist movements.

**Table 1:** Dataset description.

Leaning	Type	N	N of messages
Far-Left	Channel	34	149,457
	Group	4	140,730
QAnon	Channel	17	1,069,690
	Group	11	1,245,820
Far-Right	Channel	27	416,609
	Group	2	737,234
Total	–	94	3,759,540

We analyzed the Telegram messages through topics obtained by a Latent Dirichlet Allocation (LDA) model over weekly aggregated data. Topics were qualitatively examined to identify how the discourse employed by QAnon, far-right, and far-left communities evolved over time. All data used in our qualitative analysis is available at https://osf.io/74r8c/. We also employed a Word2Vec-based topic embedding to compute the topical similarity quantitatively across the three communities (see SI for methodological details). Word2Vec word embeddings were averaged according to the LDA results to generate topic embeddings at a community level. We present results with several model hyperparameters that reinforce our main findings in the SI and the study's online repository.

## 3 Results

**Figure 1:**
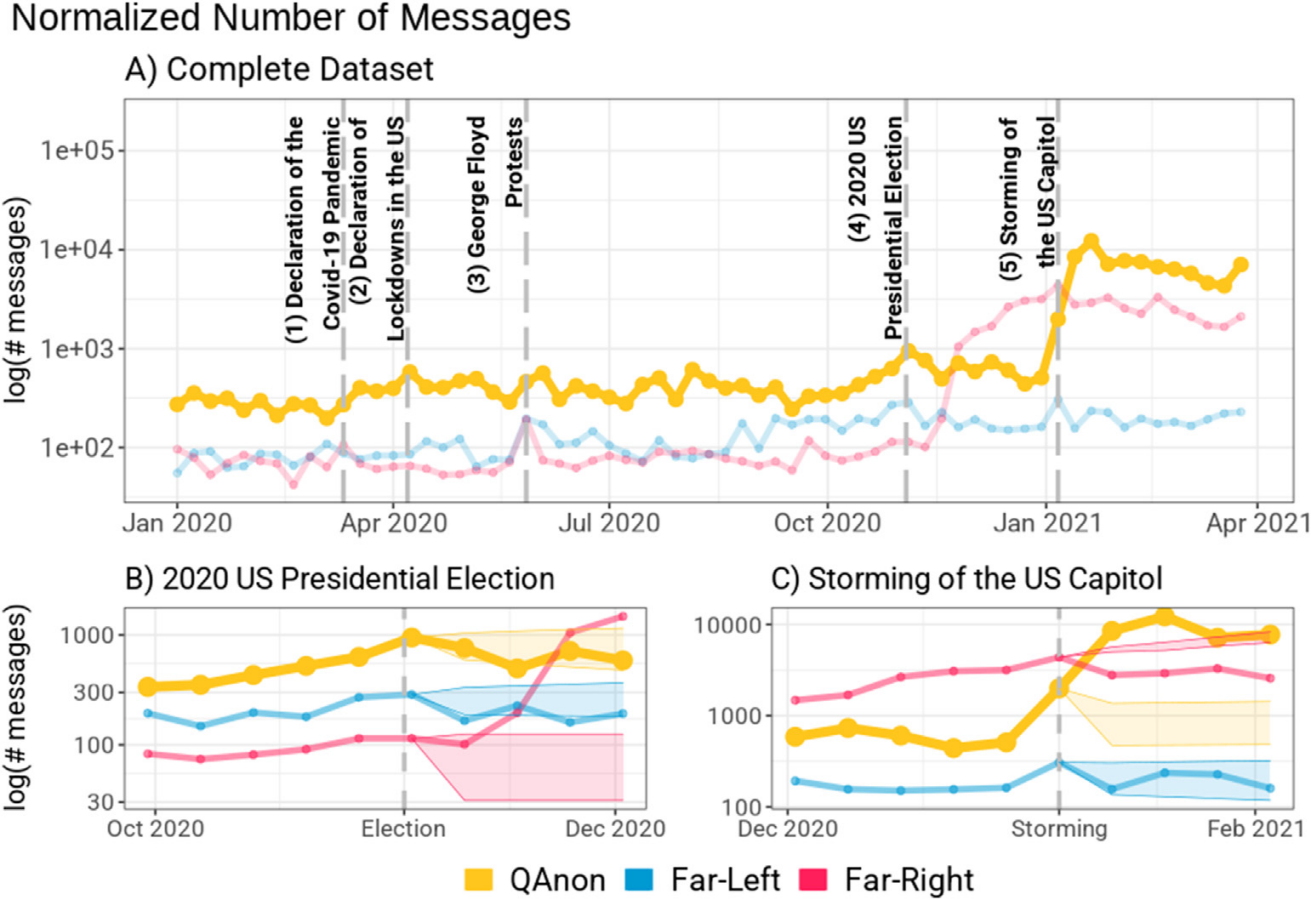
Message frequency of QAnon, far-right, and far-left communities divided by the number of groups to account for disparities in community size. We also present ARIMA predictions and their 95% confidence intervals for points after the US Presidential Election (B) and the storming of the US Capitol (C). ARIMA hyperparameters were selected based on Akaike Information Criterion (AIC) with a correction for small sample sizes (i.e., AICc). QAnon sees a gradual increase in messages leading up to the Presidential election and a significant spike in the day before and after January 6th. Far-right groups see the most significant message increase after the election and a gradual increase in the days leading to January 6th. Far-left groups remain relatively stable year-round but share a small spike with far-right groups leading up to the George Floyd protests.

The descriptive statistics of our dataset show distinctive growth patterns of the three communities. QAnon shows the largest increase in messaging over the studied period (Figure 1). The growth is substantial since the days leading up to the 2021 attack, which propelled QAnon to become the most active among the three on Telegram. In comparison, far-right communities show a pronounced spike after the Presidential election in November 2020, followed by a gradual increase leading up to the January event. There is also a small spike during the George Floyd protests. Far-left communities do not show a substantial spike in messages but like the far-right, see a small spike during the George Floyd protests. The ARIMA predictions highlight the sudden increase in messaging during these points of interest. QAnon and the far-right converged interests during the presidential election and the storming of the US Capitol, likely as a result of former US President Trump's central role. The far-right and the far-left also saw a convergence of interests during the George Floyd protests.

Qualitative analysis of the topics indicates that 'Trump'' and phrases such as “Q” and ''QAnon” identify the main topics in the QAnon communities (see SI for LDA results). For far-left communities, the recurring topics included the issue of police brutality, as identified in “police” and the names of cities where protests against police brutality occurred, such as “Minneapolis” and “Seattle.” Far-right communities were relatively reactive to the news cycle, with mentions of “vaccine,” “Trump,” and “Biden” making constant appearances throughout the year. Far-right groups also discussed race-related topics, but with a negative perspective alongside mentions of “White," “Black," “Jew,” and derogatory racial epithets. While these communities discuss popular news events, there were only a few notable times when the primary topics for QAnon were no longer Trump or conspiracy theories. Specifically, we see an increase in the number of topics related to “Biden” after the 2020 US presidential election.

**Figure 2:**
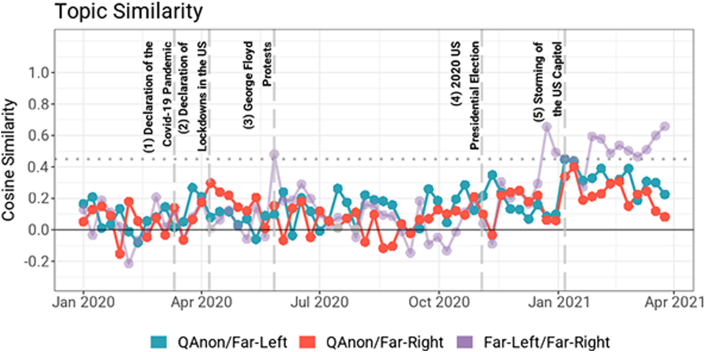
Pairwise topic similarity between QAnon, far-right, and far-left communities on Telegram. The figure presents the results considering the most important topic out of k = 5 topics returned by weekly aggregated LDA models. The dotted line represents a cosine similarity of .45, which we consider an effective barometer for topic similarity based on our qualitative analysis. Please refer to the SI for how the topics were embedded and additional results considering more topics and different values for k. Different hyperparameters did not significantly impact our analysis. Gray points are not statistically significant according to a shuffle test (see SI).

Figure 2 shows the weekly pairwise cosine similarity between topic embeddings to represent the degree to which distinct communities shared their discourse. Peaks in Figure 2 refer to when similarity is close to .45 or above on a given week. None of the peaks included QAnon, suggesting that this community's topics did not significantly overlap with the other two communities. Alongside our qualitative analysis, this notable deviation positions QAnon as a distinct movement from the far-right or the far-left.

The first prominent peak in Figure 2 appears in June 2020. Our qualitative analysis indicates the far-left and far-right communities discussed the protests following the death of George Floyd during this time. The second cohesion point between the far-right and far-left occurred in mid-December 2020. We noted an agitated debate on policing and race issues, although no singular event warranted such convergence. The mob attack on January 6 led to a heightened topical similarity across all pairs of communities, indicated by “capitol” and police-related topics. QAnon communities did not exhibit high topical similarity with either of the two other communities, yet their pairwise similarities tend to increase throughout the studied period, due to increased mentions of “Biden'' and “Trump” in the months leading to and after the 2020 US presidential election.

Table 2 presents a series of regressions that indicate how QAnon and far-right communities became marginally similar in the specific points of interest. An identical result was obtained when dividing the dataset into coronavirus-related events (i.e., events 1 and 2 in Figure 2), societal events (i.e., events 3 in Figure 2) and political events (i.e., events 4, and 5 in Figure 2). Collectively, these findings suggest that although QAnon may be a distinct political movement, it coalesces with the far-right during particular political events where Trump is a major player.

**Table 2:** Regression models of points of interests as a function of weekly pairwise topic similarities. We control for the number of weekly messages normalized by the number of groups in a community. We regressed the points of interest in Figure 2 to the pairwise cosine similarities between distinct communities to understand whether any of these major offline events correlate to a convergence between QAnon, far-right, and far-left groups. To account for the temporal dynamics, linear regressions with ARIMA errors were conducted. QAnon and far-right communities become more similar during political events where former US President Trump is a major player (i.e., events 4 and 5).

Regressions Predicting the Events of Interest				
Dependent Variable	Regression with ARIMA Errors (P = 4, Q = 1)			
	All Events	Events 1 and 2	Event 3	Events 4 and 5
QAnon/Far-Left Similarity	−0.178 (0.371)	−0.038 (0.209)	−0.315 (0.173)	0.161 (0.187)
QAnon/Far-Right Similarity	1.147∗∗∗ (0.246)	0.255 (0.194)	0.162 (0.115)	0.243∗ (0.100)
Far-Left/Far-Right Similarity	−0.106 (0.188)	0.120 (0.133)	0.473∗∗∗ (0.088)	−0.164 (0.083)
Normalized # of QAnon Messages	0.000 (0.000)	0.000 (0.000)	0.000 (0.000)	0.000∗∗ (0.000)
Normalized # of Far-Left Messages	0.000 (0.000)	−0.001 (0.000)	−0.001 (0.000)	0.001∗∗ (0.000)
Normalized # of Far-Right Messages	0.000 (0.000)	0.000 (0.000)	0.000∗∗∗ (0.000)	0.000∗∗ (0.000)
Constant	0.060 (0.044)	0.096∗ (0.044)	−0.003 (0.020)	−0.077∗∗∗ (0.017)
Observations	65	65	65	65

∗∗∗p < 0.001, ∗∗p < 0.01, ∗p < 0.05.

The number of observations refers to the number of pairwise similarities between different communities, i.e., 65 weeks from January 01, 2020 to March 31, 2021.

**Figure 3:**
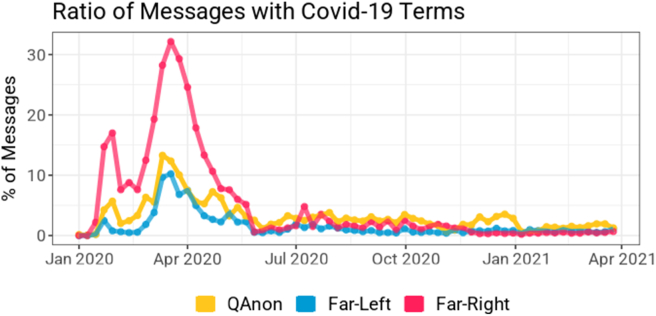
Percentage of messages mentioning the Covid-19 pandemic across QAnon, far-right, and far-left communities. The following regular expression was used to identify coronavirus-related messages: “covid|corona|virus|kung.” Covid-19 was not a major topic within QAnon and far-left communities during the studied period, whereas far-right communities mentioned the pandemic at higher rates during its first peak in the United States.

Contrary to previous work that pictured QAnon as a significant driver of Covid-19 disinformation, the pandemic rarely appeared as the most important topic for QAnon communities (see Figure 3). Only the far-right turned to Covid-19 during the height of the pandemic in the US with approximately 30% of messages dedicated to the topic. QAnon and far-left communities mentioned Covid-19 at a substantially lower rate even when the pandemic was raging in the US. The coronavirus then became a marginal topic for all communities from July 2020. Neither the declaration of the state of emergency nor state lockdowns led to a substantial increase in messaging (see Figure 1). Events related to the virus were not a strong coalescing factor to prompt a notable cosine similarly among the three communities (see Figure 2). These results were unexpected considering the number of media and academic attention given to the role of QAnon in distributing Covid-19 misinformation [34, 35].

Why QAnon topics deviate from the far-right or the far-left may be explained by the distinct information sources these political communities refer to. Media preferences influence salient topics in the community discourse [39], and such effect is evident in the topic distribution of the three Telegram communities. Messages linking information sources from other domains comprised 9.47% of the dataset. QAnon showed little similarity with far-left or far-right communities regarding the links shared, with the highest similarity being a mere 0.186 Jaccard index with the far-right communities (see SI). Links from direct media sources (i.e., everything from mainstream sources, such as the New York Times, to fringe sites, such as V-dare) were uncommon, though this also held true for the other two political communities. At first sight, these communities do not consume information from major news media outlets, though this may be a function of Telegram. Telegram does not provide news sources directly, as Facebook or Twitter, and thus would require users to go directly to the news site in order to share the news. Users of these communities seemed more inclined to find links and videos from other platforms and share the information in that manner (i.e., finding a video on YouTube and sharing its link).

A domain-level popularity analysis of each community's information sources indicates that links to mainstream outlets were nearly non-existent in the top-10 domains linked from all communities (see SI). Only the ninth most shared domain for the far-left communities was the British news site “The Guardian.” As is expected from communities on Telegram, links to other Telegram communities comprised a significant portion of the links shared. QAnon and far-right communities often spread information from alternative social media sites such as Gab, a feature that was not seen in far-left communities. Both QAnon and the far-right shared links from far-right media outlets, such as The Gateway Pundit. Links to V-dare, an explicitly white supremacist media outlet, were substantially more present in far-right communities. Far-left communities shared information from several leftist publications, such as the Jacobin magazine, and most often shared links from Reddit.

Across all three communities, YouTube and Twitter comprised a significant proportion of links shared, making up 32% of the domains. The YouTube channels and Twitter handles shared were again considerably different between the three communities. QAnon shared the most YouTube channels with ties to traditional media outlets. QAnon's most shared channels, by a sizeable margin, were from more mainstream conservative news outlets, such as Fox News and Newsmax TV (see SI). QAnon communities consumed the least diverse set of YouTube channels, with its top 10% comprising approximately 65% of the links shared. This is in contrast to the far-right and far-left communities, whose top 10% of YouTube channels comprised approximately 44% and 37.5%.

Several key differences were highlighted when identifying the top-10 Twitter handles shared by the three communities. Once again, QAnon communities showcased the least variety in their Twitter handles, such that the top 10% of handles accounted for 65% of links shared. This in contrast to far-right and far-left communities, whose top 10% of Twitter handles accounted for approximately 47% and 38% of the Twitter links, respectively. The Jaccard Index shows that QAnon exhibits little similarity with the other two communities. Interestingly, QAnon communities shared tweets from President Trump and members of his administration substantially more than the other groups (see SI).

## 4 Discussion

Our mixed-methods analyses suggest several critical differences between QAnon and other extreme political communities. Although the data indicates that QAnon's overlap with the far-right was significant during the November 2020 election and the January 6th attack, their similarity remained low. Collectively, QAnon appears to be a distinct political movement that may coalesce with existing extremist groups during major political events. This finding is supported by the non-overlap in information sources; QAnon shared links from substantially different domains, particularly within YouTube and Twitter, than both the far-right and far-left. QAnon communities showcased the least diversity in their shared links and most often referred to YouTube channels affiliated with mainstream outlets or Twitter accounts of members of the Trump cabinet. These findings, in concert, guide that QAnon communities are dissimilar to both the typical far-right and far-left movements. While its theories may be fringe, much of the information QAnon followers cite is mainstream, such as videos from traditional news outlets and Twitter accounts of public officials.

Though QAnon has grown to be an international movement, we were intentional with our focus on exclusively US-centered QAnon communities. QAnon subcultures have adapted to culturally relevant discourse, establishing relative autonomy regarding their interests [36]. Analyses of QAnon must thus be geographically specific. Rather than a global view, the Telegram dataset we examined offers a restricted yet more complete view of the US-centric QAnon communities. Regarding the far-right and far-left, we were similarly discerning in terms of geography. While some previous research has segmented the far-right movements [37] and accounted for distinctions between different sub-groups (e.g., neo-Nazi and anti-LGBTQ groups), both are understood to be far-right and thus we did not make such distinctions in our analysis [38].

Understanding that people's political beliefs motivate their media consumption, we analyzed the link shared within the communities [39]. The ideological leaning of the news sites shared by QAnon channels and groups should thus help serve as a proxy for the group's beliefs. Their most shared YouTube channels within QAnon communities, Fox News [40] and Newsmax [41], are aligned with the traditional conservative movement in the US rather than the fringe far-right. There are, however, a few exceptions; the QAnon communities on Telegram shared information sources commonly identified as far-right, such as Jake Posobiec, a host on One America News Network (OANN) with identifiable ties to white supremacists [42]. We note that the common sources for the far-right and QAnon had connections to Trump. Jake Posobiec, for instance, gained prominence when Trump retweeted him in August 2017 [43]. The far-right site OANN, another source commonly shared by the QAnon Telegram communities, was deeply popular with the Trump family nearing the end of his administration [44]. Outside of those far-right sources and individuals legitimized by the former President, much of the information shared by the QAnon community on Telegram could be identified as traditionally conservative within the contemporary political discourse.

Concerning other QAnon research, our observations support the notion that QAnon is not particularly tied to one ideological fringe camp. The movement does not cleanly identify with the various subsets of far-right ideologies, be they race-based identity movements or general hate groups. Moreover, QAnon groups do not seem to intersect with far-left movements. Our quantitative assessment showcases the activity of QAnon on Telegram and provides a more descriptive analysis of when this increased activity took place, finding that the greatest growth occurred in the days leading up to January 6, 2021. This contrasts with the far-right groups, which saw the largest increase in communication during the weeks after the November 2020 election.

Unlike other research, we identified different internal conversations in QAnon groups and those of other movements. Whereas other research found QAnon and far-right movements to share similar topics, particularly on different social media platforms, we did not find this topical similarity on Telegram. We hypothesize that this is a function of the messaging app, which allows for more siloed conversations in QAnon specific groups and channels. Unlike other platforms (e.g., Twitter), in which there exists an open universe of conversations and a flow of information from various movements and groups, Telegram allows movement-specific communication channels that may be more difficult to influence. To a certain degree, Telegram may be more representative of a movement's dialogue given the ability to both be public for members and privately contained from other groups. Future work could explore how Telegram's privacy-focused functionalities may affect extremist groups' conversations.

This is not to undersell the threat posed by QAnon. Our data reinforce the danger of QAnon by providing legitimacy to media reports pointing to the increasing number of QAnon believers on Telegram [45]. The sheer number of messages after the January attack indicates that the lack of new Q drops, the last of which was posted in December 2020, has not yet led its members to leave the community. At a bare minimum, QAnon communities are still disturbingly active months beyond any new information from Q.

What was not surprising within our dataset was the significance of the topic “Trump” and “QAnon” for the community. Unlike the far-right and the far-left, which seemed more reactive to news, “Trump” and “QAnon” consistently remained the most important weekly topic for QAnon communities. Surprisingly, Covid-19 neither came up often as the most important weekly topic for QAnon nor comprised an unexpectedly high share of messages. This stands in contrast to the far-right communities, which were especially interested in Covid-19 during the early stages of the virus in the US before moving on to other topics of interest.

Our mixed-methods analysis allowed us to better understand the conversations occurring within the QAnon movement. Challenging the widespread notion that QAnon is a branch of the far-right movement, our research offers a new perspective that this new conspiracy theory is different from commonly studied extremist movements. Interestingly, we found that QAnon has either positioned itself as a more mainstream global political movement or attracted more mainstream members to its ranks, as evidenced by the increasing mainstream content that QAnon absorbs. Unlike the far-right and the far-left that both share and consume less mainstream news, QAnon's increasing reference to mainstream conservative content should be of concern. Mainstream right-wing communities and individuals must reconcile with the role they have played and will continue to play in shaping the QAnon movement. QAnon poses a significant threat to social and political institutions, and its growth into mainstream politics and news media suggests that it may be here to stay.

## Declarations

### Author contribution statement

Saifeldeen Zihiri, Gabriel Lima: Conceived and designed the experiments; Performed the experiments; Analyzed and interpreted the data; Wrote the paper.

Jiyoung Han, Meeyoung Cha, Wonjae Lee: Conceived and designed the experiments; Wrote the paper.

### Funding statement

This work was supported by the Institute for Basic Science (IBS-R029-C2), the National Research Foundation of Korea (NRF-2017R1E1A1A01076400, NRF-2020S1A5B5A16083698), the BK21 Plus Postgraduate Organization for Content Science in Korea, and the Henry Luce Foundation.

### Data availability statement

Data associated with this study has been deposited at https://osf.io/74r8c/. Complete data and scripts for replication are available upon request.

### Declaration of interests statement

The authors declare no conflict of interest.

### Additional information

No additional information is available for this paper.

## Appendix A Supplementary data

202201_QAnon_Heliyon_Revised_R2_Clean_V2 202201_QAnon_Heliyon_Revised_R2_Clean_V2

## Footnotes

2 It is important to clarify that these definitions are unique to the United States political landscape, as our research focuses solely on the QAnon movement most tied to the United States. Our definitions are not intended to place a value judgement.
