# Supplementary material for: QAnon shifts into the mainstream, remains a far-right ally
Source: Heliyon. 2022 Jan 21;8(2):e08764. doi: 10.1016/j.heliyon.2022.e08764 (PMC8816675; doi:10.1016/j.heliyon.2022.e08764)
Supplement: 202201_QAnon_Heliyon_Revised_R2_Clean_V2 [file mmc1.docx]

**Supplementary Information**

**QAnon Shifts Into the Mainstream, Remains a Far-Right Ally**

**Methodology**

*Topic Analysis and Embedding*

Latent Dirichlet Allocation (LDA) is a generative probabilistic model for discrete data. Each item in a dataset is modeled as a finite mixture over an underlying set of topics (see (1) for all mathematical modeling). LDA identifies k topics that are represented by a distribution of the words contained in a collection of documents (i.e., a collection of Telegram messages). Each topic is composed of a distribution of all words in the dataset alongside their log-likelihood of pertaining to that topic. Each document in the dataset can be described by distribution over the k topics.

For every week w and Telegram group g with a vocabulary V_w,g_, we obtain the j-th out of k topics:

$$t_{w, g, j}= \bigcup_{i}^{V_{w, g}} \left( \omega_{i}, p_{i} \right),$$

where ω_i_ and p_i_ are word probability tuples returned by the LDA model for the j-th topic. We calculate topic importance by averaging the inference scores of each topic for all messages m_w,g_ in week w and group g:

$$importance\left( t_{w, g, j} \right)=imp\left( t_{w, g, j} \right)= \frac{\sum_{m_{w,g}\in D_{w, g}} p\left( m_{w,g} | t_{w, g, j} \right)}{\left| D_{w, g} \right|},$$

where D_w,g_ is the set of all messages in week w and group g. We qualitatively analyzed the top-10 words for each topic t_w,g,j_ from all groups in our dataset for k=3, 5, 10.

Our investigation is restricted to key topics, i.e., messages discussed by group g and week w with the highest probability. After a thorough analysis of the key topics, one of the researchers inspected all other topics and did not observe any significant distinctions from our main findings. Nevertheless, we highlight that the qualitative analysis does not cover every single subject discussed by the groups in the dataset but focuses on those topics that explained their discourses the most. The most important topic explained approximately 49.5, 36.7%, 26.3% of all topics obtained by LDA for k=3, 5, 10, respectively. We present the topic importance distribution over time in the SI for all values of k.

For comparison between groups of different political orientations (i.e., far-left, far-right, and QAnon), we obtained a topic embedding constructed from a Word2Vec embedding matrix trained on the whole dataset (2). We employed the word embedding matrix to allow for comparisons across weeks and groups. The Word2Vec matrix returns an n-dimensional vector ω_i_ for each word. We construct topic embeddings as a weighted vector of each topic’s word distribution:

$$\vec{t_{w, g, j}}= \frac{\sum_{i}^{l<\left| V_{w, g} \right|} \omega_{i}*p_{i}}{\sum_{i}^{l<\left| V_{w, g} \right|} p_{i}}.$$

We use the top-l words in each topic concerning log-likelihood for calculating the topic embedding. After a visual analysis of the log-likelihood distribution across all topics, we observed that l=50 comprised most of the topic’s content without giving much weight to words with low log-likelihood. Topic embeddings were not used in our qualitative analysis and are only used for visualization and supporting analysis. Topic embeddings did not influence the main qualitative findings presented in this paper.

We report results for k=5 in the main text. However, this analysis does not account that LDA returns k topics with different levels of importance. Thus, we also present results using a topic embedding that accounts for top-m topics returned by LDA in the SI. In this scenario, k-topic embeddings (kt) were constructed as a weighted vector of topic embeddings:

$$\vec{{kt}_{w, g}}= \frac{\sum_{j}^{m} imp\left( \vec{t_{w, g, j}} \right)* \vec{t_{w, g, j}}}{\sum_{j}^{m} imp\left( \vec{t_{w, g, j}} \right)}.$$

 The main findings of the paper are consistent regardless of which topic embeddings are used. The similarity between groups of different political orientations was calculated based on cosine similarity. For week w, we compute the pairwise similarity between communities a and b as:

$$similarity_{w}\left( a, b \right)=cosine similarity\left( \frac{\sum_{\begin{aligned} g_{a} is a group \\ of type a \end{aligned}} D_{w, g_{a}}*\vec{t_{w, g_{a}, 1}}}{\sum_{\begin{aligned} g_{a} is a group \\ of type a \end{aligned}} D_{w, g_{a}}} , \frac{\sum_{\begin{aligned} g_{b} is a group \\ of type b \end{aligned}} D_{w, g_{b}}*\vec{t_{w, g_{b}, 1}}}{\sum_{\begin{aligned} g_{a} is a group \\ of type b \end{aligned}} D_{w, g_{b}}} \right),$$

where a, b ∈ {far-right, far-left, QAnon}. We present results using k-topic embeddings using the same equation in the study’s online repository (see below).

*Link Analysis*

Links shared across all communities in the dataset were obtained through regular expressions, identifying 355,855 messages with at least one link (i.e., 9.47% of our dataset). Our analysis shows that Twitter and YouTube are the most significant web domains shared by all three communities, accounting for on average 32% of all links.

To examine Twitter hyperlinks, we utilized the user handle information that was directly identifiable in the shared URLs. An alternative approach would have been to obtain user profile information via the Twitter API, yet we found that many users whose tweets were shared on Telegram had been banned from the platform and were no longer accessible. Hence, we focused our analysis on the handles identified by the URLs.

YouTube URLs only contain a video ID. We used this video ID to request the official YouTube API for information about the video’s channel information. After identifying the channel’s ID, we obtained the channel’s name, which was later used in the qualitative analysis.

*Data Availability*

Data used for qualitative analysis is available at https://osf.io/74r8c/. Complete data and scripts for replication are available upon request. Please contact the first authors for access. This decision was made due to the harmful content included in the dataset.

**YouTube and Twitter Analysis**

Regarding YouTube, far-right communities shared a variety of different kinds of channels. This included media outlets from other countries, such as the Russian state-controlled international television network R. Far-left communities shared largely independent channels non-affiliated with a traditional news media outlet, such as the decentralized media collective known as Unicorn Riot. The only two communities that show similarities within the top ten channels shared were QAnon and right-wing groups, who shared videos uploaded by Fox News, The White House, and the Right-Side Broadcasting Network.

Regarding Twitter, far-right communities shared various handles ranging from foreign-based accounts, such as the Russian-state affiliated account Sputnik, to anti-fascist accounts. Far-left communities also had a more equitable variety of handles with an emphasis on traditional leftist Twitter accounts. QAnon and far-right communities had one major similarity by sharing many tweets by Disclose.tv. This news aggregator alleges to be non-partisan though outed as a conspiracy site and holds the number two and number one rank for the two communities, respectively. All three communities had President Trump’s Twitter account in their top ten most shared handles. The data used for analysis is available at the study’s repository: <https://osf.io/74r8c/> and below.

**Topic Analysis Results**

Topic lists are available at the study’s repository: <https://osf.io/74r8c/>. We present results for k=3, 5, 10 (i.e., the number of topics) in the weekly LDA model. Topics are ordered by order of importance according to their inference score. Topics were obtained using the *tomotopy* Python library. The repository also includes variations of Fig. 2 using k=3, 5, 10 and presenting 1) the most important topic, 2) half of the topics in order of importance, and 3) all topics. Changes in embedding do not affect the results of our finding. The repository also includes the inference score of the weekly topics.

We note a few differences between the various graphs when adjusting for the number of topics. K=3 highlights the most significant spikes as Left-Right during the George Floyd protests and the events of January 6th. K=5 has a similar spike during the George Floyd protests but no such spike on the events of January 6th. Rather, there is a more significant spike, once again among the far-right and the far-left, almost immediately before the storming of the U.S Capital.

The k=10 results are substantially different, with continuous spikes occurring before and after January 6th, again among the far-right and far-left, and a sudden QAnon/far-right spike during March 2021. It is also worth noting that k=10 includes a higher number of spikes generally, this is likely due to the increased number of secondary themes and common colloquialism being considered. Outside of these spikes, the trend for QAnon/far-right and QAnon/far-left is largely the same amongst the three graphs.

Shuffle test was used to check statistical significance in pairwise similarities. For each weekly combination of communities’ topic embeddings, we fixed one of the embeddings and calculated the similarity distribution with respect to the second embedding, after shuffling 1000 times. We repeated this procedure fixing the second topic embedding while shuffling the first. T-test was used to compute the pairwise similarity with the level of significance of .05.

**References**

1. Blei D.M., Ng A.Y., Jordan M.I., Latent dirichlet allocation, J. Mach. Learn. Res. 3 (2003) 993–1022.
2. Mikolov T., Grave E., Bojanowski P., Puhrsch C., Joulin A., Advances in pre-training distributed word representations, arXiv preprint arXiv:1712.09405 (2017).

| Rank | Far-Left | QAnon | Far-Right |
| --- | --- | --- | --- |
| 0 | reddit.com - n=22205 (40.63%) | youtube.com - n=54100 (19.26%) | youtube.com - n=10037 (16.44%) |
| 1 | twitter.com - n=12136 (22.21%) | twitter.com - n=42060 (14.97%) | telegram - n=9417 (15.42%) |
| 2 | youtube.com - n=3842 (7.03%) | telegram - n=41465 (14.76%) | twitter.com - n=5281 (8.65%) |
| 3 | telegram - n=948 (1.73%) | bitchute.com - n=9431 (3.36%) | vdare.com - n=4365 (7.15%) |
| 4 | instagram.com - n=609 (1.11%) | rumble.com - n=7208 (2.57%) | archive.is - n=3805 (6.23%) |
| 5 | en.wikipedia.org - n=412 (0.75%) | gab.com - n=4871 (1.73%) | bitchute.com - n=1464 (2.40%) |
| 6 | jacobinmag.com - n=402 (0.74%) | bit.ly - n=4095 (1.46%) | instagram.com - n=781 (1.28%) |
| 7 | theintercept.com - n=241 (0.44%) | instagram.com - n=3698 (1.32%) | archive.vn - n=730 (1.20%) |
| 8 | theguardian.com - n=223 (0.41%) | thegatewaypundit.com - n=3694 (1.31%) | thegatewaypundit.com - n=491 (0.80%) |
| 9 | ift.tt - n=218 (0.40%) | facebook.com - n=2614 (0.93%) | dailymail.co.uk - n=438 (0.72%) |
| 10 | facebook.com - n=217 (0.40%) | usadramalert.com - n=1606 (0.57%) | rumble.com - n=417 (0.68%) |
| 11 | truthout.org - n=211 (0.39%) | breitbart.com - n=1191 (0.42%) | vm.tiktok.com - n=392 (0.64%) |
| 12 | nytimes.com - n=189 (0.35%) | whitehouse.gov - n=1102 (0.39%) | boards.4chan.org - n=373 (0.61%) |
| 13 | en.m.wikipedia.org - n=172 (0.31%) | dailymail.co.uk - n=1068 (0.38%) | zerohedge.com - n=352 (0.58%) |
| 14 | itsgoingdown.org - n=170 (0.31%) | qmap.pub - n=1016 (0.36%) | dlive.tv - n=327 (0.54%) |
| 15 | bit.ly - n=169 (0.31%) | thebl.tv - n=1016 (0.36%) | nypost.com - n=324 (0.53%) |
| 16 | vice.com - n=164 (0.30%) | foxnews.com - n=996 (0.35%) | rt.com - n=309 (0.51%) |
| 17 | newrepublic.com - n=125 (0.23%) | zerohedge.com - n=949 (0.34%) | bitwave.tv - n=303 (0.50%) |
| 18 | scmp.com - n=124 (0.23%) | theepochtimes.com - n=906 (0.32%) | breitbart.com - n=284 (0.47%) |
| 19 | crimethinc.com - n=117 (0.21%) | nypost.com - n=845 (0.30%) | en.m.wikipedia.org - n=270 (0.44%) |

Table S1. Top-20 domains shared on Telegram within QAnon, far-left, and far-right communities.

|  | Far-Left | QAnon | Far-Right |
| --- | --- | --- | --- |
| Far-Left | 1 | - | - |
| QAnon | 0.084 | 1 | - |
| Far-Right | 0.110 | 0.186 | 1 |

Table S2. Jaccard index (i.e., similarity between sets) between domains shared on Telegram within QAnon, far-left, and far-right communities.

| Rank | Far-Left | QAnon | Far-Right |
| --- | --- | --- | --- |
| 0 | @i - n=94 (0.77%) | @realdonaldtrump - n=1851 (4.40%) | @disclosetv - n=143 (2.71%) |
| 1 | @iwriteok - n=72 (0.59%) | @disclosetv - n=1230 (2.92%) | @sputnikint - n=120 (2.27%) |
| 2 | @realdonaldtrump - n=71 (0.59%) | @danscavino - n=949 (2.26%) | @breaking911 - n=94 (1.78%) |
| 3 | @joshuapotash - n=69 (0.57%) | @breaking911 - n=809 (1.92%) | @donaldjtrumpjr - n=80 (1.51%) |
| 4 | @protest_nyc - n=57 (0.47%) | @i - n=700 (1.66%) | @jackposobiec - n=54 (1.02%) |
| 5 | @anoncatanoncat - n=50 (0.41%) | @vincentcrypt46 - n=450 (1.07%) | @pataltscotland - n=52 (0.98%) |
| 6 | @itsa_talia - n=49 (0.40%) | @jackposobiec - n=383 (0.91%) | @i - n=49 (0.93%) |
| 7 | @crimethinc - n=44 (0.36%) | @secpompeo - n=354 (0.84%) | @realdonaldtrump - n=48 (0.91%) |
| 8 | @happyroadkill - n=44 (0.36%) | @donaldjtrumpjr - n=327 (0.78%) | @gwensnyderphl - n=46 (0.87%) |
| 9 | @igd_news - n=40 (0.33%) | @karluskap - n=327 (0.78%) | @rt_com - n=36 (0.68%) |
| 10 | @ur_ninja - n=40 (0.33%) | @toddwal47945939 - n=267 (0.63%) | @nickjfuentes - n=35 (0.66%) |
| 11 | @vitalistint - n=39 (0.32%) | @trumpwarroom - n=250 (0.59%) | @danscavino - n=34 (0.64%) |
| 12 | @tariqnasheed - n=35 (0.29%) | @thebias_news - n=235 (0.56%) | @mrandyngo - n=32 (0.61%) |
| 13 | @ashagony - n=31 (0.26%) | @jamesokeefeiii - n=234 (0.56%) | @richardgrenell - n=27 (0.51%) |
| 14 | @griffinmalone6 - n=31 (0.26%) | @jsolomonreports - n=219 (0.52%) | @baronstrucker - n=23 (0.44%) |
| 15 | @thescoopusa - n=30 (0.25%) | @cbs_herridge - n=216 (0.51%) | @michellemalkin - n=22 (0.42%) |
| 16 | @theonion - n=30 (0.25%) | @llinwood - n=212 (0.50%) | @elijahschaffer - n=21 (0.40%) |
| 17 | @fg5vbftxbx - n=30 (0.25%) | @riseandriseaga2 - n=201 (0.48%) | @ell_tsnmi - n=20 (0.38%) |
| 18 | @mrolmos - n=30 (0.25%) | @inevitable_et - n=196 (0.47%) | @columbiabugle - n=19 (0.36%) |
| 19 | @blacksocialists - n=29 (0.24%) | @deptofdefense - n=188 (0.45%) | @godwins123456 - n=19 (0.36%) |

Table S3. Top-20 Twitter handles shared on Telegram within QAnon, far-left, and far-right communities.

|  | Far-Left | QAnon | Far-Right |
| --- | --- | --- | --- |
| Far-Left | 1 | - | - |
| QAnon | 0.027 | 1 | - |
| Far-Right | 0.031 | 0.063 | 1 |

Table S4. Jaccard index (i.e., similarity between sets) between Twitter handles shared on Telegram within QAnon, far-left, and far-right communities.

| Rank | Far-Left | QAnon | Far-Right |
| --- | --- | --- | --- |
| 0 | Reza Rezaie Khanghah - n=40 (1.12%) | Fox News - n=1815 (4.60%) | Lemuel - n=254 (3.08%) |
| 1 | Jonas Čeika - CCK Philosophy - n=29 (0.81%) | Newsmax TV - n=991 (2.51%) | Azərbaycan Respublikası Müdafiə Nazirliyi - n=86 (1.04%) |
| 2 | Philosophy Tube - n=28 (0.79%) | Monkey Werx US - n=523 (1.33%) | Fox News - n=79 (0.96%) |
| 3 | Unicorn Riot - n=27 (0.76%) | Right Side Broadcasting Network - n=510 (1.29%) | The Golden One - n=74 (0.90%) |
| 4 | Perseus999 - n=25 (0.70%) | The White House - n=460 (1.17%) | RT - n=52 (0.63%) |
| 5 | Thought Slime - n=24 (0.67%) | One America News Network - n=350 (0.89%) | Ruptly - n=51 (0.62%) |
| 6 | Jreg - n=22 (0.62%) | GEORGEnews - n=329 (0.83%) | The White House - n=40 (0.49%) |
| 7 | The Intercept - n=22 (0.62%) | Quantum Love & Truth #5DConsciousness - n=302 (0.77%) | Right Side Broadcasting Network - n=38 (0.46%) |
| 8 | The Gravel Institute - n=20 (0.56%) | Fox Business - n=299 (0.76%) | Mista Anonymous - n=37 (0.45%) |
| 9 | Democracy At Work - n=19 (0.53%) | Universe Inside You - n=287 (0.73%) | RATMVEVO - n=36 (0.44%) |
| 10 | The Hill - n=17 (0.48%) | Our Everyday Lives - n=281 (0.71%) | Grift Patrol - n=33 (0.40%) |
| 11 | Renegade Cut - n=17 (0.48%) | Gina Maria Colvin Hill - n=263 (0.67%) | NBC News - n=30 (0.36%) |
| 12 | LastWeekTonight - n=17 (0.48%) | QNews Official TV - n=256 (0.65%) | PBS NewsHour - n=30 (0.36%) |
| 13 | All Gas No Brakes - n=16 (0.45%) | Mustang Medic - n=233 (0.59%) | Acrion - n=28 (0.34%) |
| 14 | The New Centre for Research & Practice - n=16 (0.45%) | Project Veritas - n=223 (0.57%) | Zachary Denman - n=28 (0.34%) |
| 15 | Then & Now - n=16 (0.45%) | BardsFM - n=210 (0.53%) | Patriotische Jugend - n=27 (0.33%) |
| 16 | WeAreChange - n=16 (0.45%) | Sky News Australia - n=207 (0.52%) | RickAstleyVEVO - n=26 (0.32%) |
| 17 | China Uncensored - n=15 (0.42%) | THE TRUTH - n=195 (0.49%) | Ashton Daniel - n=25 (0.30%) |
| 18 | Vocal Synthesis - n=15 (0.42%) | Scotty Mar10 - n=188 (0.48%) | C-SPAN - n=25 (0.30%) |
| 19 | LacanOnline - n=14 (0.39%) | Trump White House Archived - n=183 (0.46%) | Rob Bliss - n=25 (0.30%) |

Table S5. Top-20 YouTube channels shared on Telegram within QAnon, far-left, and far-right communities.

|  | Far-Left | QAnon | Far-Right |
| --- | --- | --- | --- |
| Far-Left | 1 | - | - |
| QAnon | 0.023 | 1 | - |
| Far-Right | 0.042 | 0.081 | 1 |

Table S6. Jaccard index (i.e., similarity between sets) between YouTube channels shared on Telegram within QAnon, far-left, and far-right communities.
